# A general method to predict optical rotations of chiral molecules from their structures

**DOI:** 10.1039/d2ra08290j

**Published:** 2023-02-06

**Authors:** Hai-Feng Ji

**Affiliations:** a Department of Chemistry, Drexel University Philadelphia PA 19104 USA hj56@drexel.edu +1-215-895-1265 +1-215-895-2562

## Abstract

The relationship of the chiroptical response of a molecule to its absolution configuration does not exist now. In this letter, I intend to report a general rule with exceptions to predict the sign of optical rotation of chiral molecules with a RCHXY structure from their absolute configurations using the Hammett constant, *σ*_p_, which is based on the electron withdrawing/donating power of functional groups. In this rule, a priority list of functional groups based on the electron withdrawing powers of the groups are used. When the lowest priority group is in the back of the molecule, a clockwise arrangement of the other three priorities from the most electron withdrawing to the least withdrawing (1-2-3) is predicted to be dextrorotatory, the counterclockwise arrangement is predicted to be levorotatory.

## Abstract 2

The relationship of the chiroptical response of a molecule to its absolution configuration does not exist now.

## Graphical abstract

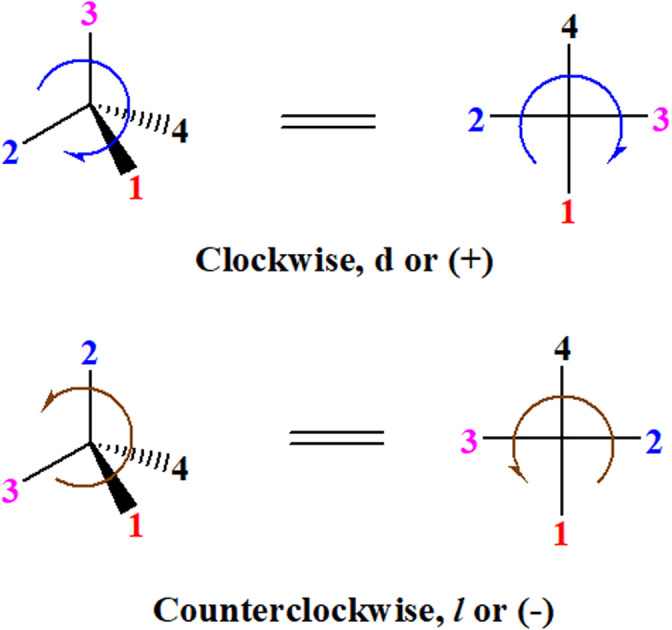

## Introduction

2022 marks the 200th birthday of Louis Pasteur, who separated the first pair of chiral molecules in the 19th century.^1^ Since then, scientists have strived to make a connection between the optical rotation of a chiral molecule and its molecular structure. Unfortunately, the relationship of the chiroptical response of a molecule to its absolution configuration has still not been achieved or does not exist now. The famed Cahn–Ingold–Prelog (CIP) rules^2^ have been used to assign the absolute R and S configurations around a stereocenter, but every textbook of organic chemistry specifically addresses that there is no correlation between the R/S configuration and the optical rotation of chiral molecules. Experiments must be conducted in order to determine the optical properties of chiral molecules. Currently, much of the effort in this field has focused on computational chemistry to improve the accuracy in predicting the optical rotation from the structures of molecules.^3–6^ It is doubtful that such a correlation would ever exist. However, there is likely to be a method that is close to a general rule to predict the optical rotations of chiral molecules with some exceptions.

Chiral molecules are those that have no plane of symmetry and can rotate linearly polarized light. In a simple chiral molecule with only one chiral carbon, four different groups are bonded to carbon so it can be considered a spiral object. A linearly polarized light beam can be regarded as a superposition of two circularly polarized electromagnetic waves.^7^ They proceed in the same frequency and direction but with the opposite rotation. The spiral shape of chiral molecules enables them to have an opposite effect on the two circularly polarized electromagnetic waves, so the two waves travel at slightly different speeds through the chiral medium,^8^*i.e.*, they no longer have the same phase on the original plane. This phase shift results in the cancellation of the two waves at a slightly different plane, causing an optical rotation (or deviation) of the linearly polarized light beam.

Since the rotation of the linearly polarized light beam is caused by the spatial arrangement of the four groups, it is expected that parameters related to the electron characteristics of the four groups could be used to correlate the optical rotation of chiral molecules with their absolute configurations. In this letter, I intend to report a general rule with exceptions to predict the optical rotation sign of chiral molecules from their absolute configurations using the order of the electron-withdrawing/donating powers of the four groups.

The electron withdrawing/donating properties of substituents have been used to compare the reactivities of various organic reactions, such as hydrolysis,^9^ ionization,^10^ esterifications,^11^ brominations,^12^ electrophilic aromatic substitutions,^13^*etc.* The electron-withdrawing power of functional groups follows the following order in general, but some of them shift slightly depending on reactions:NR_2_ < NH_2_ < OH < OR < NHCOR < OCOR < R < Ph < CH=CR_2_ <H < X < CHO < COR < COOR < COOH < COCl < CF_3_ < C=N < SO_3_H < NO_2_ <NH_3_^+^ < NR_3_^+^

This order can be quantitively described using various substituent constants, such as the Hammett constant,^8^ and constants used in the Swain–Lupton equation,^14^ the Taft equation,^15^ the Grunwald–Winstein equation,^16^ and the Yukawa–Tsuno equation,^17^*etc.* Among these, the Hammett equation was the first developed and has been widely used, and the Hammett constants are a set of the most complete list of substituent constants.

The Hammett constants, *σ*_p_ and *σ*_m_, contain both inductive and resonance effects of substituents on the hydrolysis of benzoic acid esters. *σ*_p_ has less effect from the steric hindrance because the substituents are on the para position than that of *σ*_m_ at the meta position. The applications of these constants are far beyond the reactions only.^18^ Both *σ*_p_ and *σ*_m_ have been used in this study to predict the optical rotation from the structures of molecules. *σ*_p_ constants are used in this report because they have a better performance, suggesting the steric effect is not critical for the correlation between the optical rotation and the absolute configuration. *σ*_p_ constants of some major organic groups are listed in Table 1. Stronger electron-withdrawing groups have a greater positive number, and electron donating groups have negative numbers. All the numbers are obtained from two ref. 18 and 19.

Hammett constants, *σ*_p_, of some major organic functional groups. Note: when the constant is close to zero of H, the constant may not be accurately used to predict the optical response of chiral molecules. The originally reported *σ*_p_ Hammett constants of phenyl groups in Table 1 is −0.01. This constant has been arbitrarily changed to 0.01 for the prediction of optical responses of chiral molecules with higher accuracy based on a large number of compounds that contain phenyl group and H group

**Table:** 

	NMe_2_	NH_2_	OH	OR(OMe)	NHCOCH(Me)_2_	OCOR(Me)	R(Me)	H
*σ*_p_	−0.83	−0.66	−0.37	−0.27	−0.1	−0.19	−0.17	0

**Table:** 

	Ph	X (Cl, Br)	COOR(H)	COCl	CF_3_	CN	NO_2_	NR_3_^+^
*σ*_p_	0.01	0.23	0.45	0.61	0.54	0.66	0.78	0.82

**Fig. 1:**
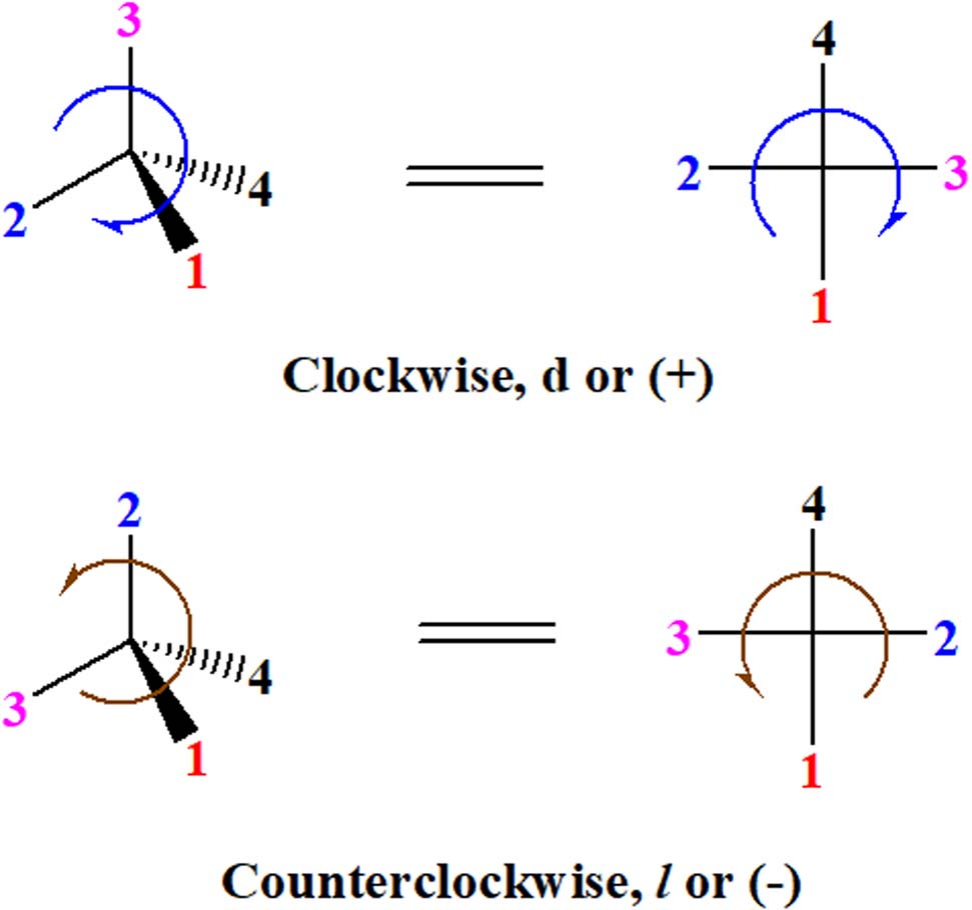
The 3D structure and fisher projection of the general rule. Electron withdrawing powers 1 > 2> 3 > 4.

A general rule on predicting the chiroptical sign of chiral molecules can be described as: first, assign the priorities to each bonded group surrounding the stereocenter based on their electron-withdrawing power (1, most to 4, least). Second, move the lowest priority (4) group to the back of the molecule. Count the other three priorities from the most electron-withdrawing to the least withdrawing (1-2-3). A clockwise is predicted to be dextrorotatory, the counterclockwise is predicted to be levorotatory. In this rule, instead of a priority list of functional groups based on the atomic number of the elements, electron-withdrawing powers of the groups are used. The structure can be written either as a 3D structure or a fisher projection (Fig. 1).

Two model systems were studied in this work to confirm how the above general rule applies to the prediction of the chiroptical response (sign) of chiral molecules from their molecular structures and the accuracy rate. The first is an RCHXY system. The second is the 20 l-amino acids in their acidic forms.

## Discussion

Table 2 shows the prediction result of RCHXY molecules, where R is CH_3_ as those listed in the table, but other alkyl groups gave the same results on the sign, X and Y are two other popular organic functional groups. Several common functional groups, such as –OCOR, –NO_2_ were not included in the table since there are no chiroptical data on these chemicals. The group NH_3_^+^ will be specifically discussed in the amino acid section later. The rotation signs of these compounds were obtained from literature or available chemical catalogs. Those signs that could not be found were listed as —.

**Table:** Prediction of optical rotations of RCHXY molecules using the electron-withdrawing powers of the four groups. The numbers next to the groups are the *σ*_p_ constants

X	Y					
	–NH_2_	–OH	–OR	–OCOR	–Ph	–X
–NH_2_ or –NR_2_	n/a	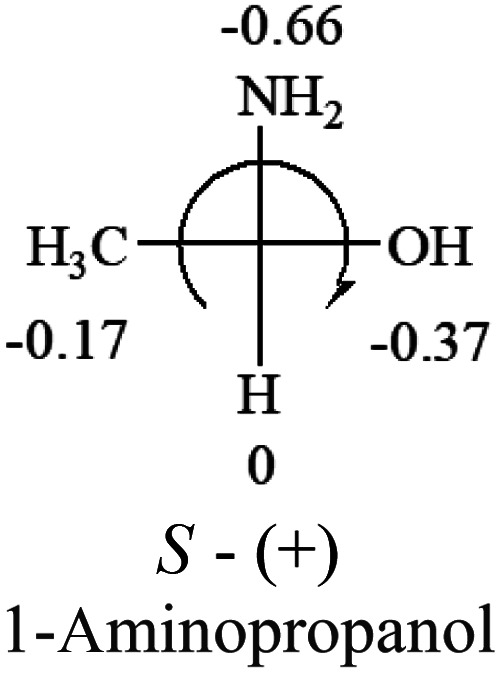	—	—	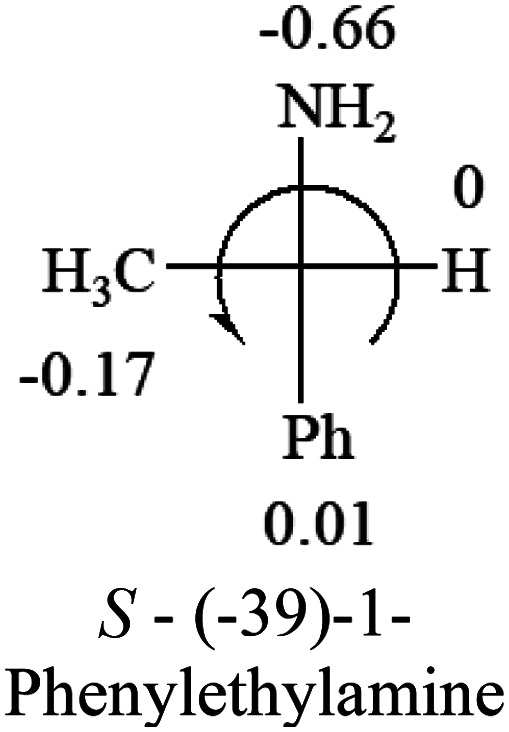	—
–OH or –OR	See row 2-column 3	—	—	—	See row 4-column 3	—
–Ph	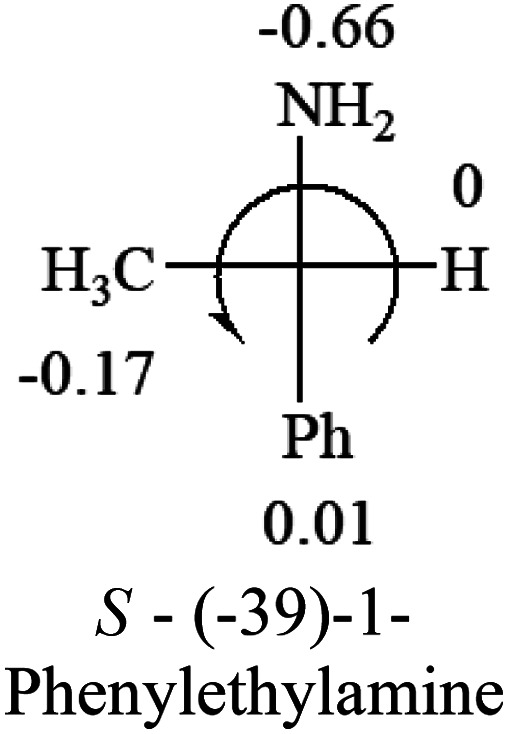	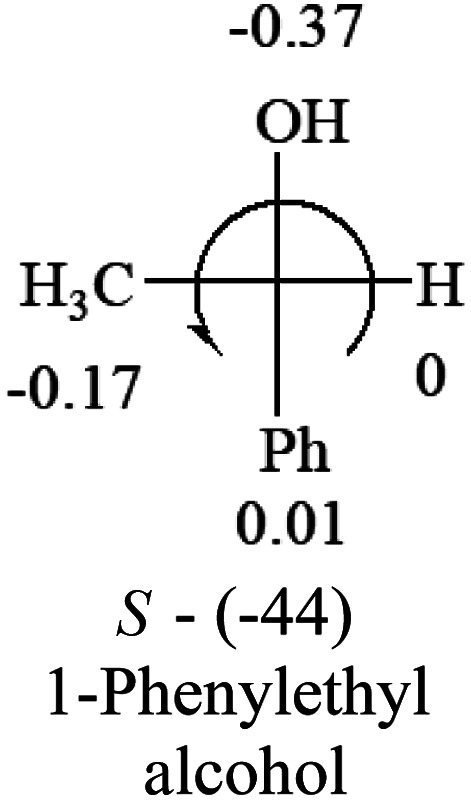	—	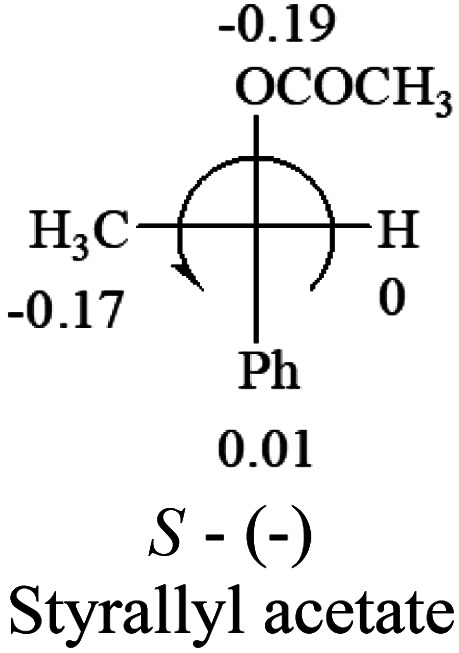	—	—
–COOR or –COOH	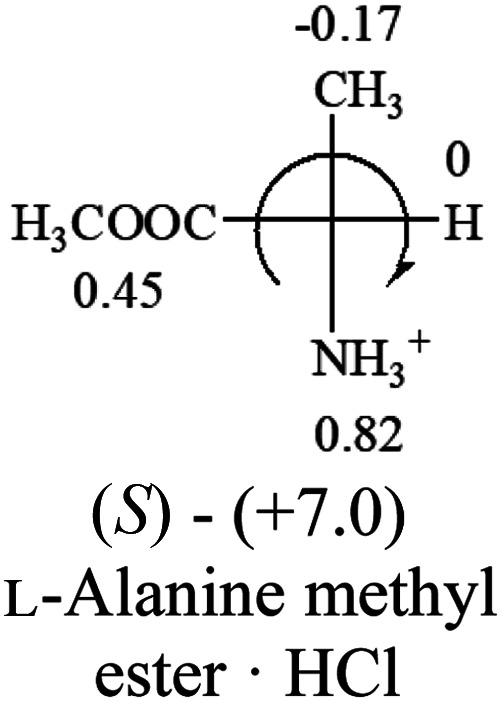	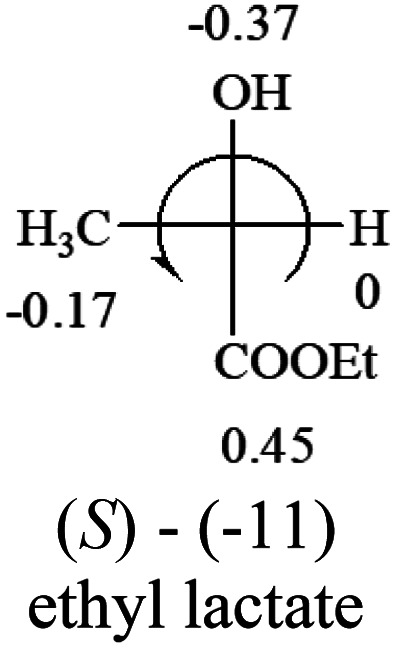	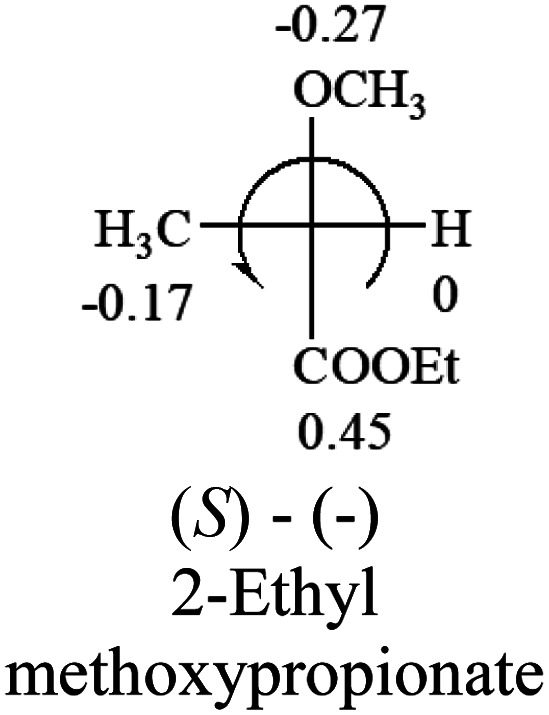	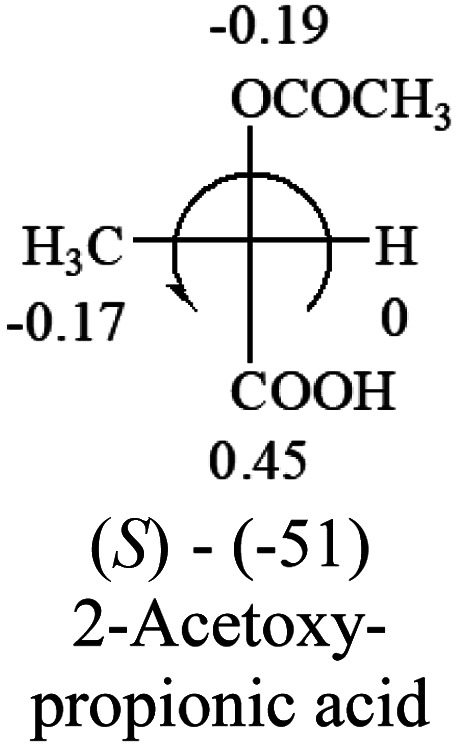	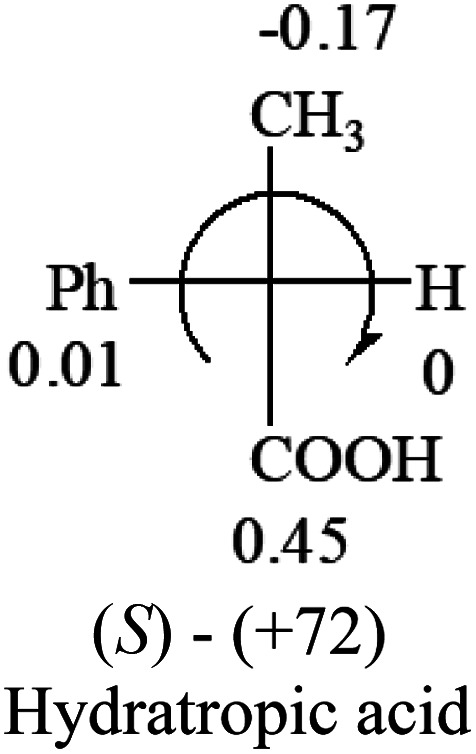	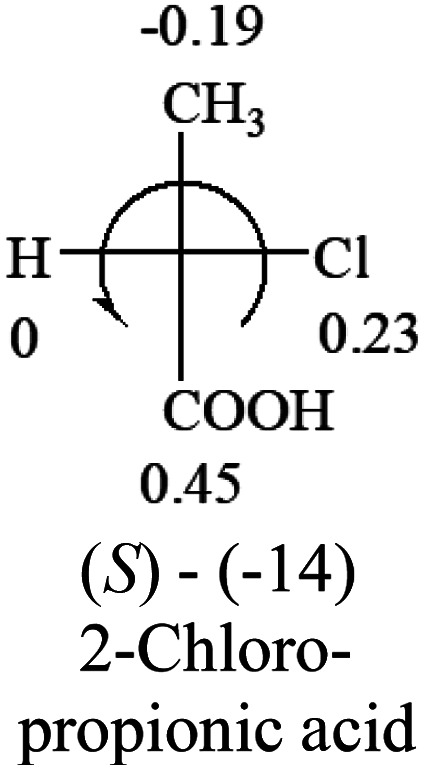
–CF_3_	—	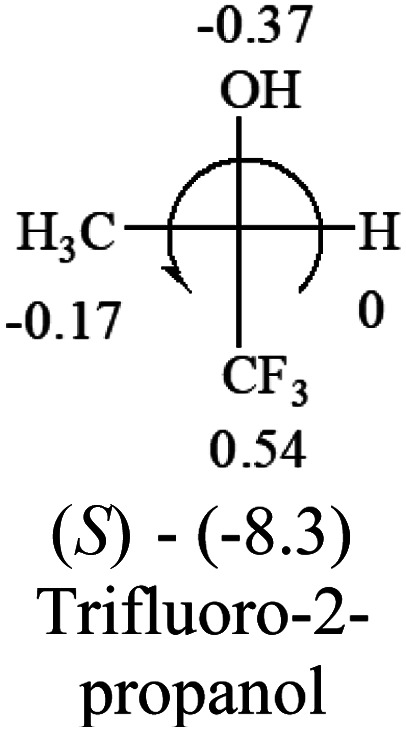	—	—	—	—
–CN	—		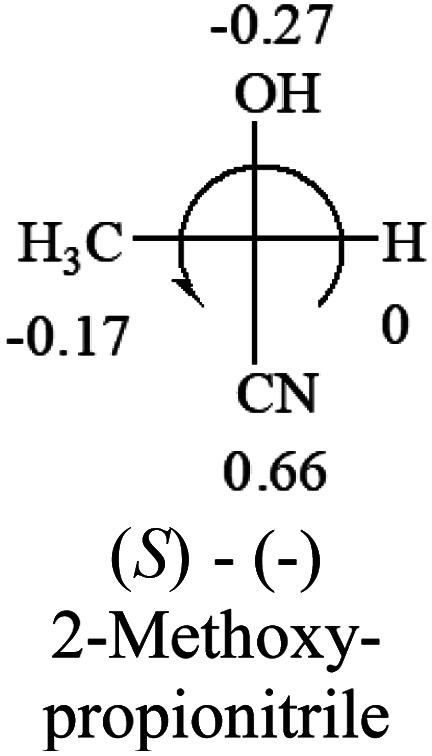	—	—	—

The result shows that all the molecules listed in Table 2, except for lactic acid (*S*, +2.6), obey the electron-withdrawing rule above, indicating a high accuracy in predicting the chiroptical sign of chiral molecules from their molecular structures. It is not unexpected that lactic acid disobeys the rule since the dominating species in water is its dimmer and larger self-aggregation *via* hydrogen bonding over its monomer form,^20^ which will change the electron-withdrawing powers of both –OH and –COOH groups. As a comparison, lactic acetate ethyl ester doesn't form such aggregation in water and its monomer form does obey the rule. The chiroptical rotation of lactate is included in Table 2 to show the general trend since the –COOEt in lactate has the same Hammett constant as that of –COOH in lactic acid.

**Fig. 2:**
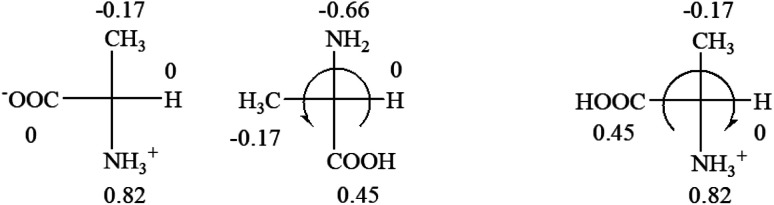
Left: optical rotation sign predicted from l-(*S*)-alanine (CH_3_CHNH_2_COOH). Right: sign predicted from the protonated form of l-(*S*)-alanine (CH_3_CHNH_3_^+^COOH).

All the 20 natural l-amino acids, except for glycine and cysteine, have the S absolute configuration, but the signs of specific rotation can be both positive and negative. In the early 20th century, Lutz and Jirgensons^21^ reported that when a strong acid was added to an aqueous solution of natural l-amino acids, the specific rotation of the protonated amino acids became more positive than their zwitterionic forms, which is called the CLJ rule.^22^ This phenomenon has been used in industry due to its reliability, but no explanation has been proposed. The above prediction of the optical rotations of chiral chemicals can well explain this phenomenon. Taking l-(*S*)-alanine as one example, in pure water, it is in equilibrium with its zwitterionic form (eqn (1)), and this mixture of alanine has a negative sign, *i.e.*, levorotary. According to the prediction method set in this work, the original form of alanine (CH_3_CHNH_2_COOH) is expected to have a levorotary sign since the order of the electron-withdrawing powers is counterclockwise from –COOH to –H and to –CH_3_ (Fig. 2 left). The zwitterionic form may not contribute to the optical rotation of alanine since the constants of both –COO^−^ and H are zero. The addition of HCl protonates the carboxylate group (eqn (2)),^23^ as a result, the most electron withdrawing group in alanine changes from COOH to NH_3_^+^, and the sign of this protonated form is positive since the order of the electron-withdrawing powers is now clockwise from –NH_3_^+^ to –COOH and to –H (Fig. 2 right).

It is noteworthy that although the Hammett constant *σ*_p_ of –COO^−^ is zero, this constant specifically for the purpose of predicting the chiroptical response of chiral molecules can be estimated from the optical rotations of amino acids in alkaline solutions. It is known that the addition of an access amount of NaOH in the aqueous solution of alanine decreases the rotation to a more negative number, *i.e.* rotate to the further left.^20^ In the alkaline solution, alanine only exists in its anionic form, CH_3_CHNH_2_COO^−^. According to the prediction rule in this work, in order for CH_3_CHNH_2_COO^−^ to be levorotatory, the *σ*_p_ of COO^−^ should be slightly less than 0 of –H, but greater than −0.17 of –CH_3_.

1 CH_3_CHNH_2_COOH → CH_3_CHNH_3_^+^COO^−^

2 CH_3_CHNH_3_^+^COO^−^ + HCl → CH_3_CHNH_3_^+^COOH

Since the specific rotations of amino acids in pure water were recorded from a mixture of the two forms, the protonated form is used in this work to analyze how the general rule applies to the prediction of chiroptical response (sign) of l-amino acids from their molecular structures and the accuracy rate.

The result in Table 3 shows that all the protonated natural l-amino acids, except for phenylalanine, obey the general rule introduced in this work. The exception for phenylalanine may be caused by the π-cation interaction of the benzene ring with –NH_3_^+^, which decreases the Hammett constant of –NH_3_^+^.

**Table:** Prediction of optical rotations of l-amino acids in their protonated forms. Note: cysteine: no *σ*_p_ on –CH_2_SH, but it should be <0 (–CH_2_OH); lysine: no *σ*_p_ on –(CH_2_)_4_NH_3_^+^, –(CH_2_)_3_N^+^(Me)_3_ (−0.01) was used instead; methionine: no *σ*_p_ on –CH_2_CH_2_SCH_3_. It will be close to –CH_2_CH_2_CH_3_ (−0.15); tryptophan: no *σ*_p_ on the –CH_2_-ring. It will be close to –CH_2_CH=CH_2_ (−0.14); glutamine: no *σ*_p_ for CH_2_CH_2_CONH_2_. It will be close to CH_2_CH_2_COOH (−0.07); histidine: no *σ*_p_ on –CH_2_-ring. It will be close to –CH_2_CH=CH_2_ (−0.14)

l-Alanine (+13)	l-Arginine (+21.6)	l-Asparagine (+34)	l-Aspartic acid (+24.4)	l-Cysteine (+7.9)
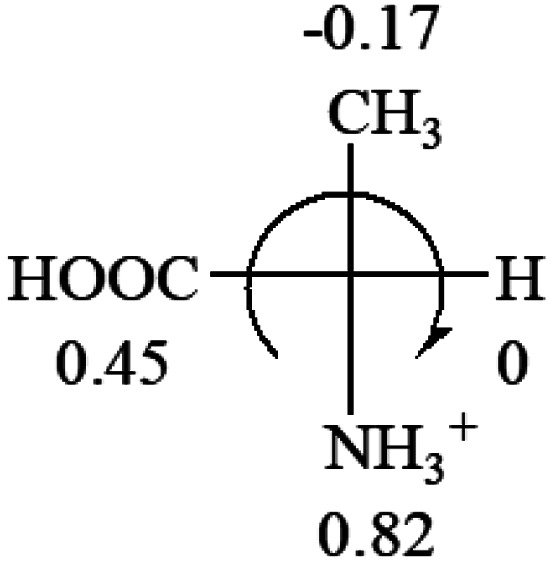	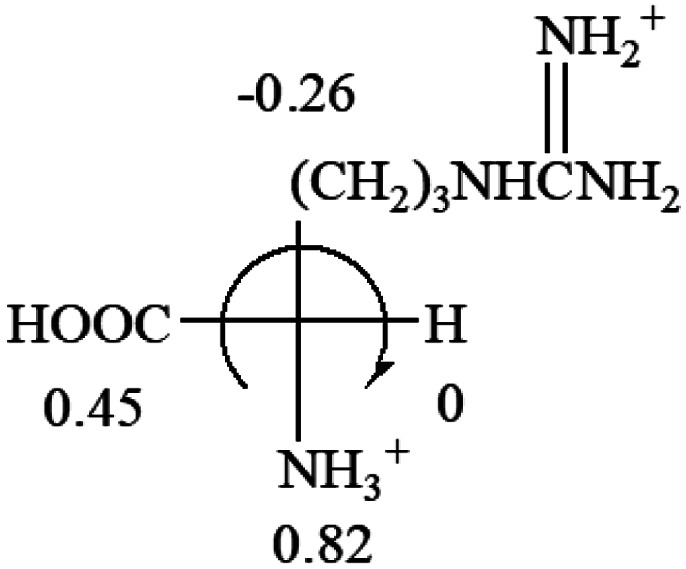	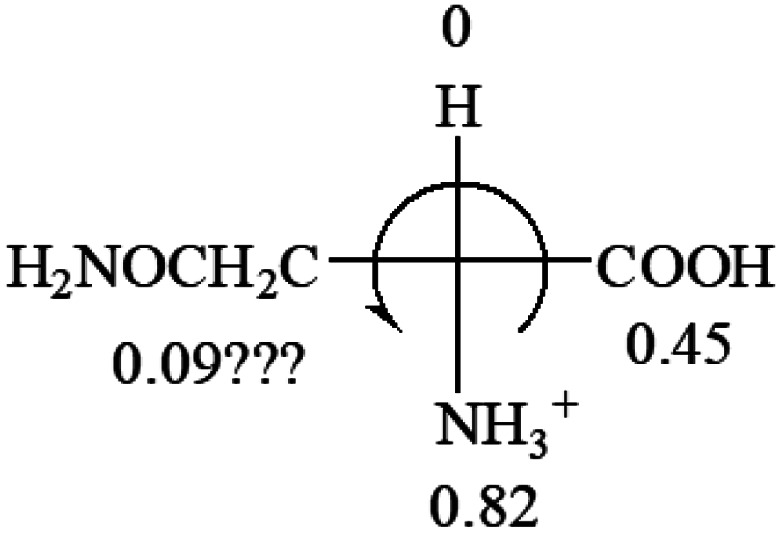	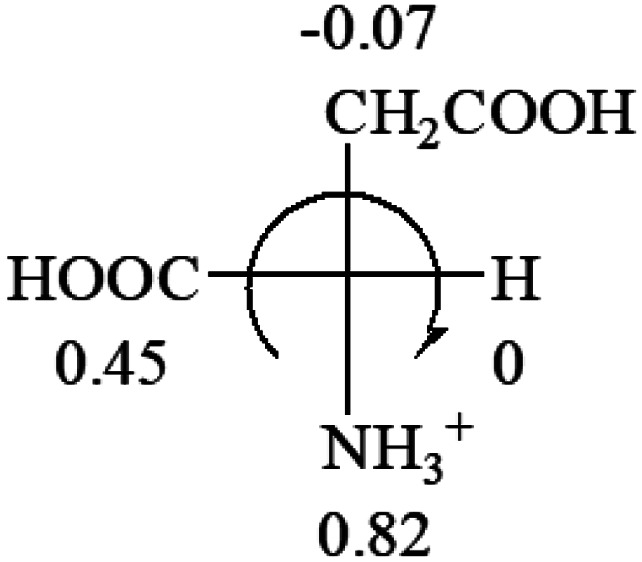	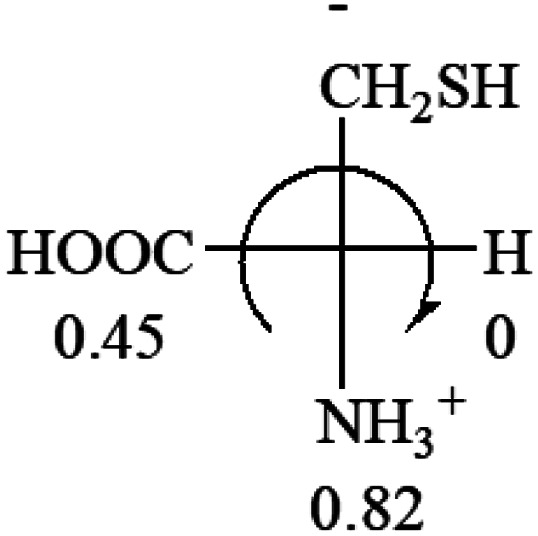
l-Glutamine (+6.5)	l-Glutamic acid (+31.5)	Glycine (not chiral)	l-Histidine (+13)	l-Isoleucine (+51.8) two chiral centers
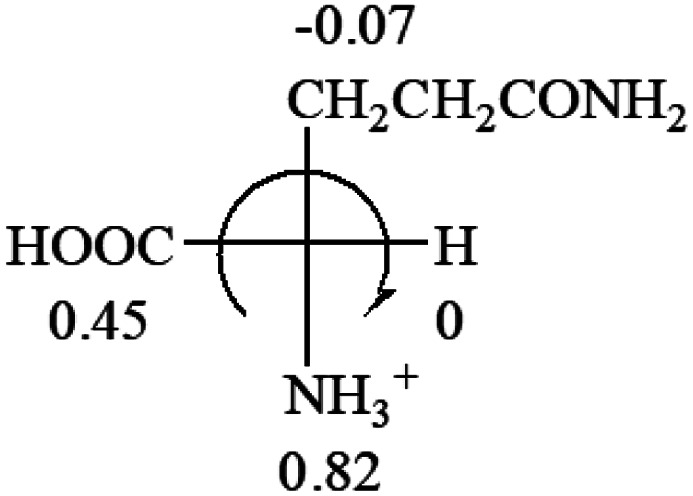	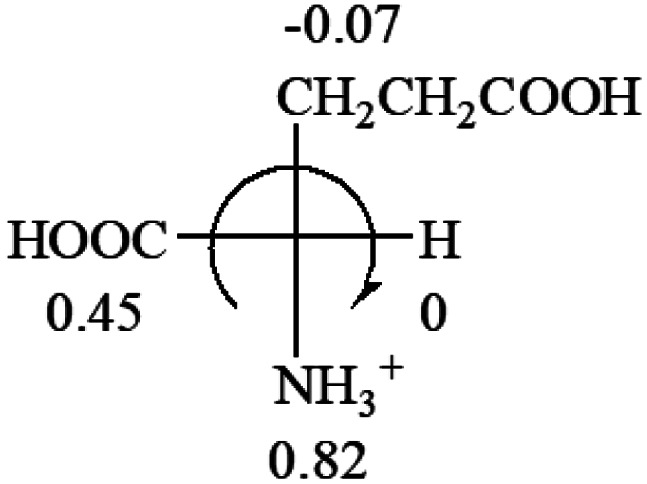	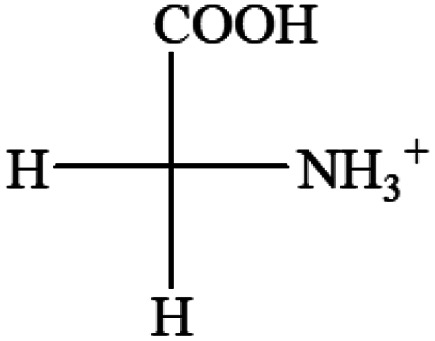	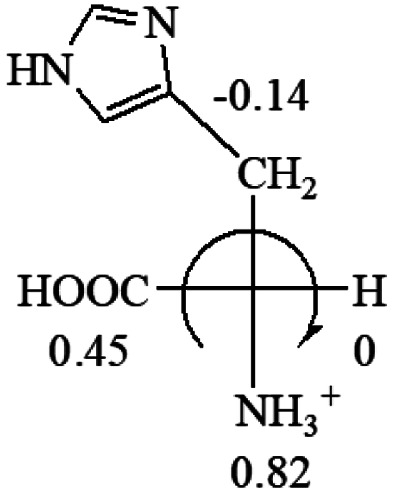	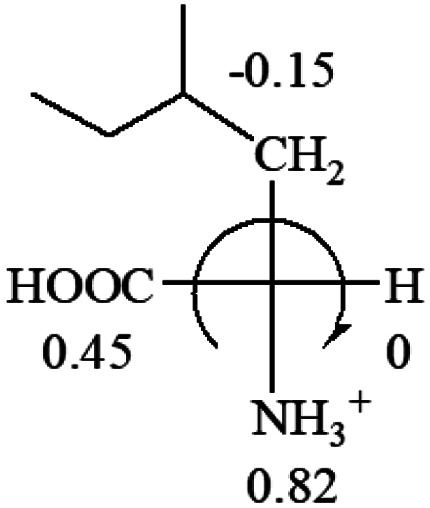
l-Leucine (+14.9)	l-Lysine (+20.5)	l-Methionine (22.8)	l-Phenylalanine (−7.4)	l-Proline (−69)
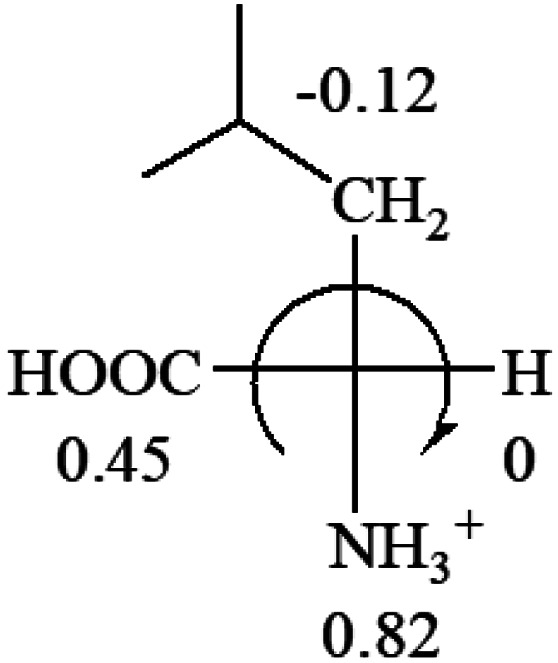	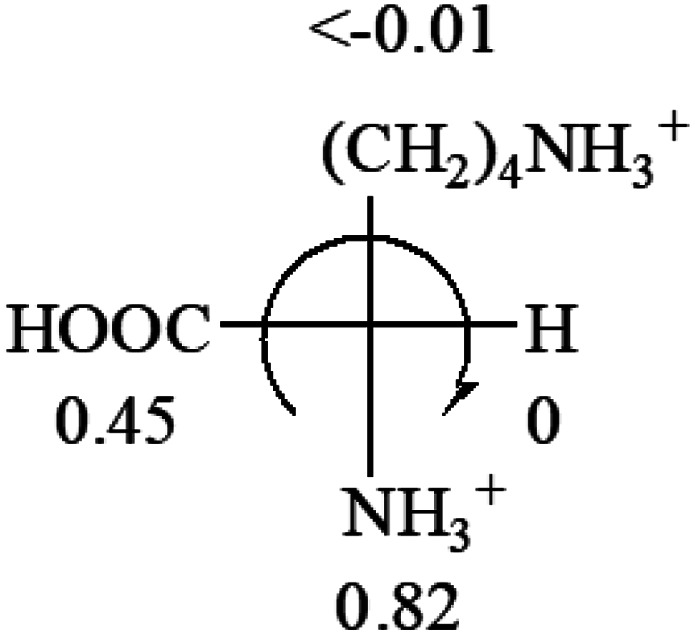	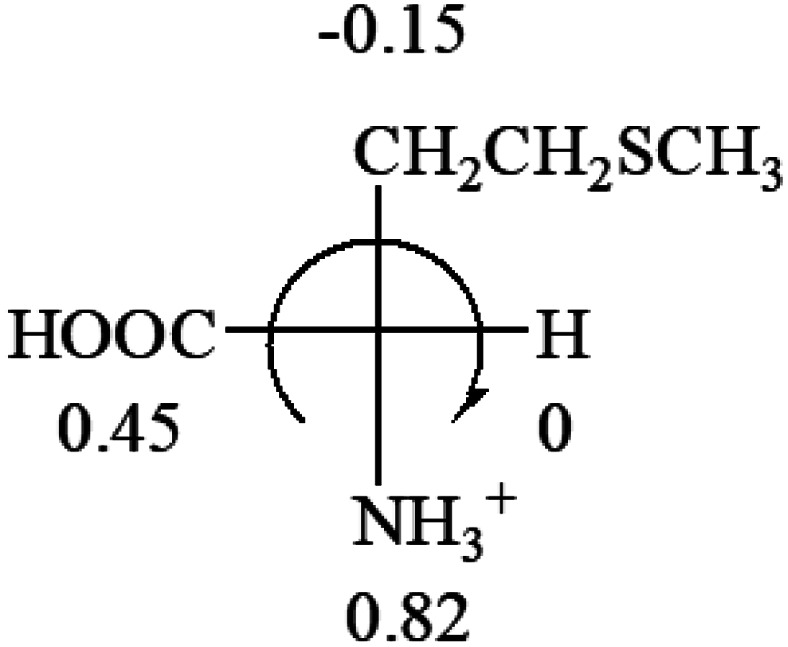	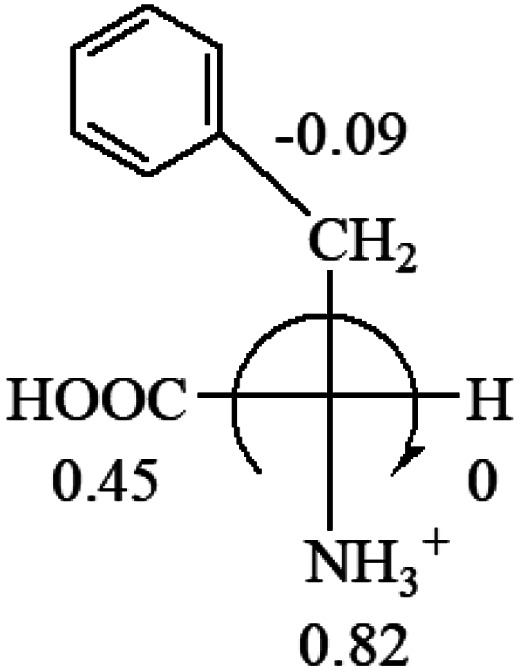	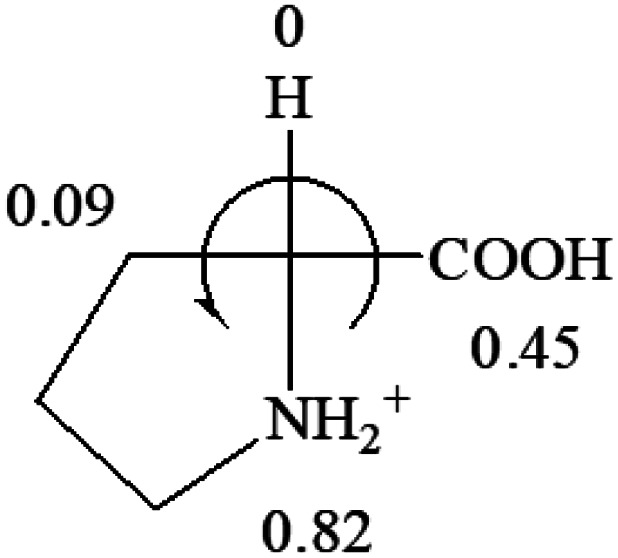
l-Serine (+15.9)	l-Threonine (**−**17.9)	l-Tryptophan (+13)	l-Tyrosine (−10.1)	l-Valine (+26.9)
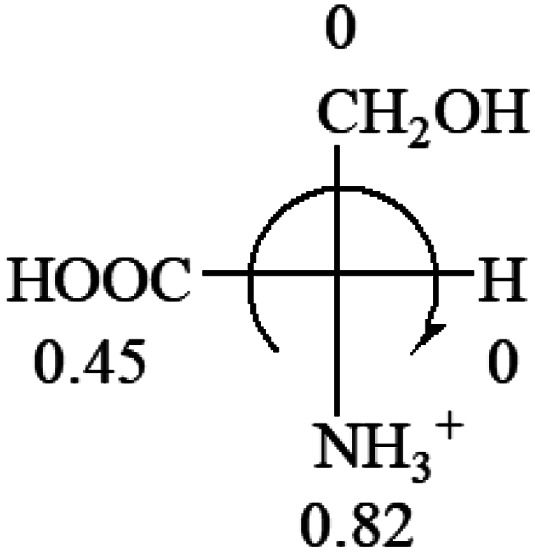	Two chiral centers	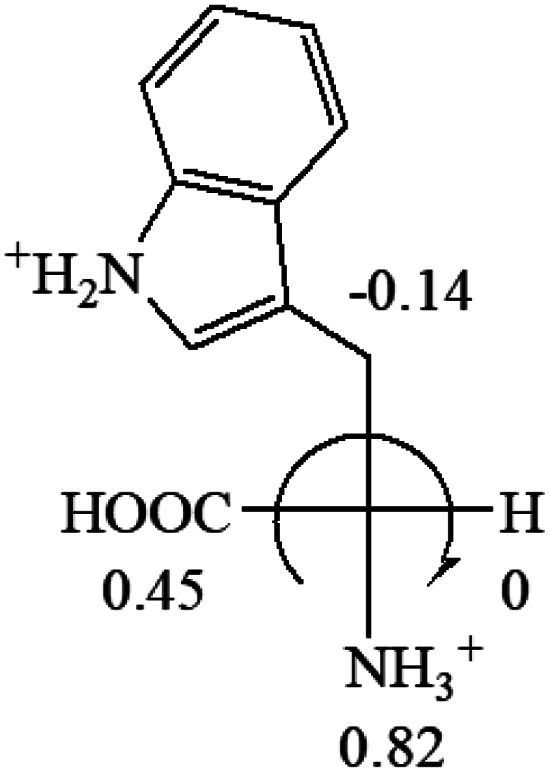	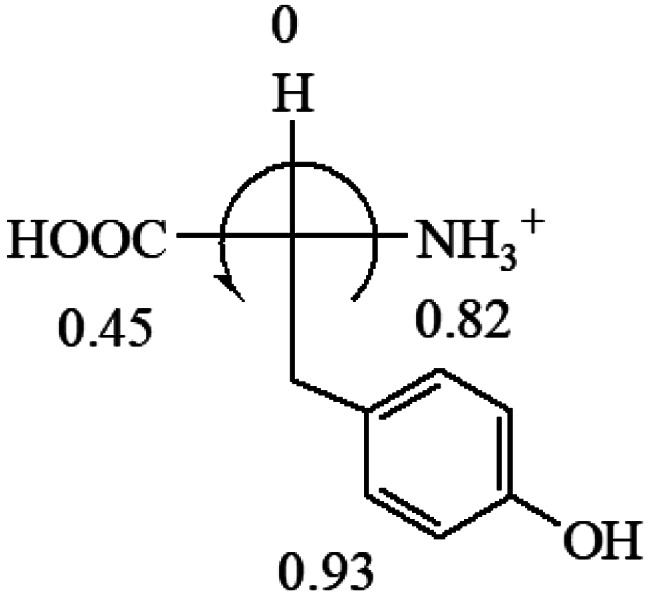	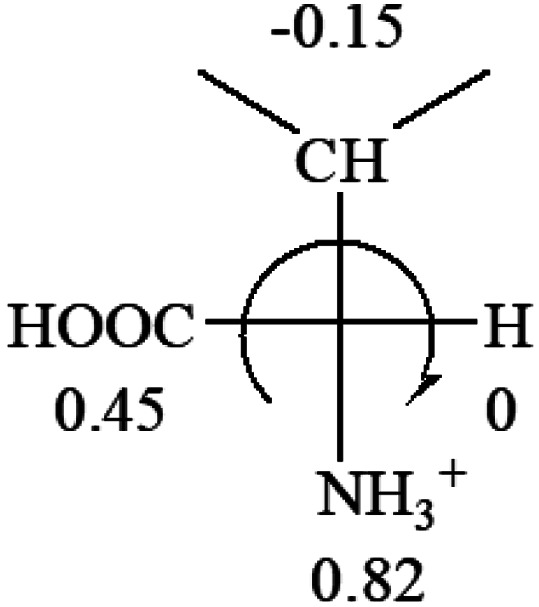

As observed in all these cases, the result showed high accuracy in predicting the chiroptical sign of chiral molecules from their molecular structures, but there are chiral molecules that do not obey the above general prediction rule. One feature in common is the existence of inter and intra- molecular interactions, such as those in lactic acid and phenylalanine. Another feature that is common, but not specifically discussed in the above examples, is that the Hammett constants of at least two groups are close to each other, such as two alkyl groups. For instance, the constants of several alkyl groups are: Me (−0.17), Et (−0.15), butyl (−0.15), isobutyl (−0.15), *etc.* The prediction of these molecules is difficult and their disagreement with experimental measurements of the optical rotation is expected. For accurate prediction of the optical rotation, the difference in *σ*_p_ between any two groups, Δ*σ*_p_, should be greater than a threshold number, such as 0.05. If Δ*σ*_p_ < 0.05, even the weak interactions of the R groups with other functional groups on the asymmetric carbon become a crucial factor to determine the optical rotation of the molecule since a minor change in the electron donating/withdrawing power of R will change the optical rotation of the molecule. Other than interactions with adjacent groups, additional structural information related to the conformation of alkyl groups must also be considered, which is known to affect the magnitude and sign of optical rotations of chiral molecules.^24^ If both features, interactions and Δ*σ*_p_, exist in one molecule, the prediction will become more challenging. For example, in CH_3_CHNH_2_CH_2_OH, the *σ*_p_ of –CH_2_OH is 0, which is the same as –H (0), and it forms internal hydrogen bonding with –NH_2_. For molecules like all of these, the quantum theoretical calculation is again needed to aid in more accurate prediction of their chiroptical responses, which might explain why such prediction did not exist so far.

In conclusion, prediction of the sign of optical rotations of chiral molecules from their absolute configurations can generally be achieved using the Hammett constants, *σ*_p_, based on the electron-withdrawing/donating power of functional groups. The Hammett constants, *σ*_p_, may further be fine-tuned specifically for the purpose of such prediction. The modification will be based on a large number of available data on chiral molecules. Re-measurement of some chemicals may be necessary to obtain accurate optical rotations, such as a change of pH to measure the optical rotation of a single form of amine or carboxylic acid species. Once this set of data is built, it is also likely to deduce a general empirical equation to calculate the amplitude of specific rotations using a data fitting method. More importantly, the general rule based on the withdrawing/donating powers of functional groups may be applied in quantum theoretical calculations to accurately calculate the sign and amplitude of specific rotations of chiral molecules in the future, especially for molecules with small Δ*σ*_p_ of two groups, or more than two chiral carbons and chiral molecules without chiral carbons.

## Conflicts of interest

There are no conflicts to declare.

## Supplementary Material

## References

[cit1] Pasteur L. , Lecons de Chlmie en 1860, Alembic Club Reprint, 1848, vol. 14

[cit2] Cahn R. S., Ingold C. K., Prelog V. (1966). Specification of Molecular Chirality. Angew. Chem., Int. Ed..

[cit3] Polavarapu P. L. (2007). Renaissance in chiroptical spectroscopic methods for molecular structure determination. Chem. Rec..

[cit4] Turbomole A. R., Bar M., Haser M., Horn H., Kolmel C. (1989). Electronic structure calculations on workstation computers: The program system turbomole. Chem. Phys. Lett..

[cit5] Crawford T. D. (2006). Ab initio calculation of molecular chiroptical properties. Theor. Chem. Acc..

[cit6] Stephens P. J., Devlin F. J., Cheeseman J. R., Frisch M. J. (2001). Calculation of Optical Rotation Using Density Functional Theory. J. Phys. Chem. A.

[cit7] Jenkins F. A. and White H. E., Fundamentals of Optics, McGraw-Hill, New York, 4th edn, 1976

[cit8] Fresnel A. , Mémoire sur la double réfraction que les rayons lumineux éprouvent en traversant les aiguilles de cristal de roche suivant les directions parallèles à l'axe, in Oeuvres complètes d'Augustin Fresnel, ed. H. de Senarmont, E. Verdet and L. Fresnel, 1866, vol. 1, pp. 731–51, translated as Memoir on the double refraction that light rays undergo in traversing the needles of quartz in the directions parallel to the axis, Zenodo, 4745976, 2021

[cit9] Hammett L. P. (1937). The Effect of Structure upon the Reactions of Organic Compounds. Benzene Derivatives. J. Am. Chem. Soc..

[cit10] Ko H. C., O'Hara W. F., Hu T., Hepler L. G. (1964). Ionization of Substituted Phenols in Aqueous Solution. J. Am. Chem. Soc..

[cit11] Taft R. W. (1952). Polar and steric substituent constants for aliphatic and o-benzoate groups from rates of esterification and hydrolysis of esters. J. Am. Chem. Soc..

[cit12] Toullec J., El-Alaoui M. (1985). Ring substituent effects on acetophenone dimethyl acetal formation. 2 Dual-parameter treatment of kinetic data for acid-catalyzed acetal formation and hydrolysis in methanol containing small amounts of water. J. Org. Chem..

[cit13] Norman R. O. C. and Coxon J. M., Principles of Organic Synthesis, CRC Press, 3rd edn, 1993, pp. 353–354, ISBN 9780748761623

[cit14] Swain C. G., Lupton E. C. (1968). Field and resonance components of substituent effects. J. Am. Chem. Soc..

[cit15] Taft R. W. (1952). Linear Free Energy Relationships from Rates of Esterification and Hydrolysis of Aliphatic and Ortho-substituted Benzoate Esters. J. Am. Chem. Soc..

[cit16] Grunwald E., Winstein S. (1948). The Correlation of Solvolysis Rates. J. Am. Chem. Soc..

[cit17] Yasuhide Y., Yuho T. (1959). Resonance Effect in Hammett Relationship. II. Sigma Constants in Electrophilic Reactions and their Intercorrelation. Bull. Chem. Soc. Jpn..

[cit18] Hansch C., Leo A., Taft R. W. (1991). A survey of Hammett substituent constants and resonance and field parameters. Chem. Rev..

[cit19] Perrin D. D. , Dempsey B. and Serjeant E. P., pKa prediction for organic acids and bases, Springer, Dordrecht, 1981, ISBN: 978-94-009-5883-8

[cit20] Kundrat M. D., Autschbach J. (2006). Time dependent density functional theory modeling of chiroptical properties of small amino acids in solution. J. Phys. Chem. A.

[cit21] Lutz I. O., Jirgensons B. (1930). Über eine neue Methode der Zuteilung optisch-aktiver α-Aminosäuren zur Rechts- oder Linksreihe I Mitteil. Ber. Dtsch. Chem. Ges..

[cit22] Greenstein J. P. and Winitz M., Chemistry of the Amino Acids, John Wiley & Sons, New York, 1961

[cit23] Kundrat M. D., Autschbach J. (2006). Time Dependent Density Functional Theory Modeling of Specific Rotation and Optical Rotatory Dispersion of the Aromatic Amino Acids in Solution. J. Phys. Chem. A.

[cit24] Pecul M., Ruud K., Rizzo A., Helgaker T. (2004). Conformational Effects on the Optical Rotation of Alanine and Proline. J. Phys. Chem. A.

